# Bone marrow metastasis of colon cancer as the first site of recurrence: A case report

**DOI:** 10.3892/ol.2014.2581

**Published:** 2014-10-01

**Authors:** DO HYOUNG LIM, SOON IL LEE, KEON WOO PARK

**Affiliations:** Division of Hematology-Oncology, Department of Medicine, Dankook University College of Medicine, Cheonan, South Chungcheong 330-715, Republic of Korea

**Keywords:** bone marrow metastasis, colon cancer, recurrence, thrombocytopenia

## Abstract

Bone marrow metastasis from solid tumors is usually accepted as not only incurable, but as fatal. Colon cancer is a relatively rare malignancy that involves the bone marrow, and to the best of our knowledge, there have been no studies in the literature reporting only bone marrow metastasis of colon cancer as the first presentation of relapse. The present study reports the case of a 74-year-old female patient treated by resection and adjuvant chemotherapy for stage IIIc colon cancer who presented with severe thrombocytopenia with intracranial hemorrhage, and the bone marrow was first and only site of metastasis. There was no evidence of skeletal metastasis. The clinical course was extremely aggressive and the patient succumbed ten days after admission, finally being diagnosed in the postmortem examination. The present study also discusses bone marrow metastasis of solid tumors, with particular respect to the diagnostic difficulties of such rare cases.

## Introduction

Bone marrow metastasis from solid tumors is usually accepted as not only incurable, but as fatal. Lung, breast and prostate malignancies in adults, and neuroblastoma and rhabdomyosarcoma in children are the most common non-hematological malignancies to frequently involve the bone marrow (1). However, almost all types of malignancy can metastasize to the bone marrow and these are occasionally reported (1–3). The frequency of detection depends on the stage of the disease and the other sites of the metastases.

Colon cancer is a relatively rare malignancy that involves the bone marrow (2,4), and to the best of our knowledge, there have been no studies in the literature reporting only bone marrow metastasis of colon cancer as the first presentation of relapse. The present study reports the case of a patient with bone marrow metastasis from colon cancer as the site of recurrence, presnting with severe thrombocytopenia and fatal intracranial hemorrhage. Written informed consent was obtained from the family of the patient.

## Case report

**Figure 1:**
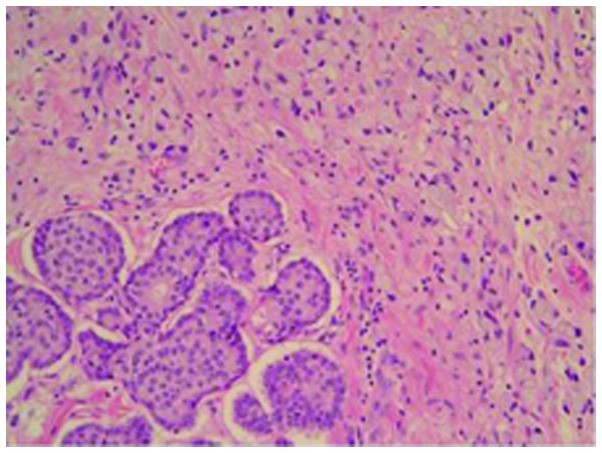
Biopsy specimen showing poorly-differentiated adenocarcinoma with signet ring cell features in the cecum (hematoxylin and eosin; magnification, ×200).

A 74-year-old female patient visited the Department of Hematology-Oncology, Dankook University of Medicine (Cheonan, Korea) for the evaluation and treatment of thrombocytopenia and an increased bleeding tendency. The patient had previously undergone a right hemicolectomy with ileocolostomy due to colon cancer three years previously. At that time, the obtained surgical specimen was diagnosed as adenocarcinoma, with signet ring cell features (Fig. 1), and the surgical staging was pT3N2M0, stage IIIc. The patient received adjuvant chemotherapy with oxaliplatin (85 mg/m^2^, day 1), leucovorin (200 mg/m^2^, days 1 and 2) and 5-fluorouracil (400 mg/m^2^ i.v. bolus and subsequently 600 mg/m^2^ i.v. continuously for 22 h, days 1–2) every two weeks for six months. Subsequent to this, the patient was followed up regularly and there was no evidence of disease recurrence until the last abdominopelvic computed tomography (CT) scans, 50 days prior to the current admission.

The patient was treated by the traditional herbal medicine clinic (Chinese Medicine Clinic, Oriental Hospital of Daejeon University, Cheonan, Korea) for approximately three weeks due to back pain that occurred from a fall. The patient was prescribed herbal medicine and treated with acupuncture on the back. One day prior to visiting Dankook University of Medicine, the patient suddenly developed a headache, dizziness, vomiting and severe thrombocytopenia, with a count of 5,000/μl, decreased from the count of 111,000/μl measured by the initial laboratory test at the clinic. The patient was therefore transferred to Dankook University of Medicine for a transfusion, and for the evaluation and treatment of the thrombocytopenia.

The initial laboratory data recorded in Dankook University of Medicine determined a white blood cell count of 5,910/μl (normal range, 4,000–10,000/μl), a hemoglobin level of 6.7 g/dl (normal range, 12–16 g/dl), a platelet count of 4,000/μl (normal range, 130,000–400,000/μl), an aspartate transaminase-alanine transaminase ratio of 45/8 IU/l (normal range, 4–37/4–41 IU/l), a total bilirubin level of 1.43mg/dl (normal range, 0.2–1.2 mg/dl), an alkaline phosphatase level of 262 IU/l (normal range, 35–104 IU/l) and a protein/albumin ratio of 5.9/3.7 g/dl (normal range, 6.4–8.3/3.4–4.8 g/dl). The prothrombin time (international normalized ratio) was 18.2 sec (1.50; normal range, 9–13 sec/0.7–1.2 international normalized ratio) and the partial thromboplastin time was 36.8 sec (normal, 25–37 sec). At the time of admission, CT scans of the brain revealed acute subdural hemorrhage on each fronto-parieto-temporal area. The patient was clinically diagnosed with drug-induced thrombocytopenia owing to herbal medicine and was initially transfused with packed red blood cells and platelet concentrates. Hematoma removal and decompression surgery was planned for after an increase in the platelet count, however, the thrombocytopenia did not improve despite the patient discontinuing the suspected herbal medicine and even though a repeated transfusion of platelet concentrates was performed and treatment with systemic steroid was administered. A bone marrow aspiration and biopsy were performed, and the patient was also injected with human immunoglobulin intravenously, however, the bone marrow aspirate showed no adequate marrow particles or cellular elements. Even though all treatment options were attempted, the platelet count had decreased to 1,000/μl, the patient’s mental status was worsening and there was an increasing amount of hemorrhage on follow-up CT scans. The Department of Neurosurgery therefore decided that an emergency procedure was necessary regardless of the risk of extreme bleeding, and the probable high morbidity and mortality. However, the patient’s family did not consent to the surgery, and following a lack of response to the medical treatment, the patient succumbed 10 days after admission.

**Figure 2:**
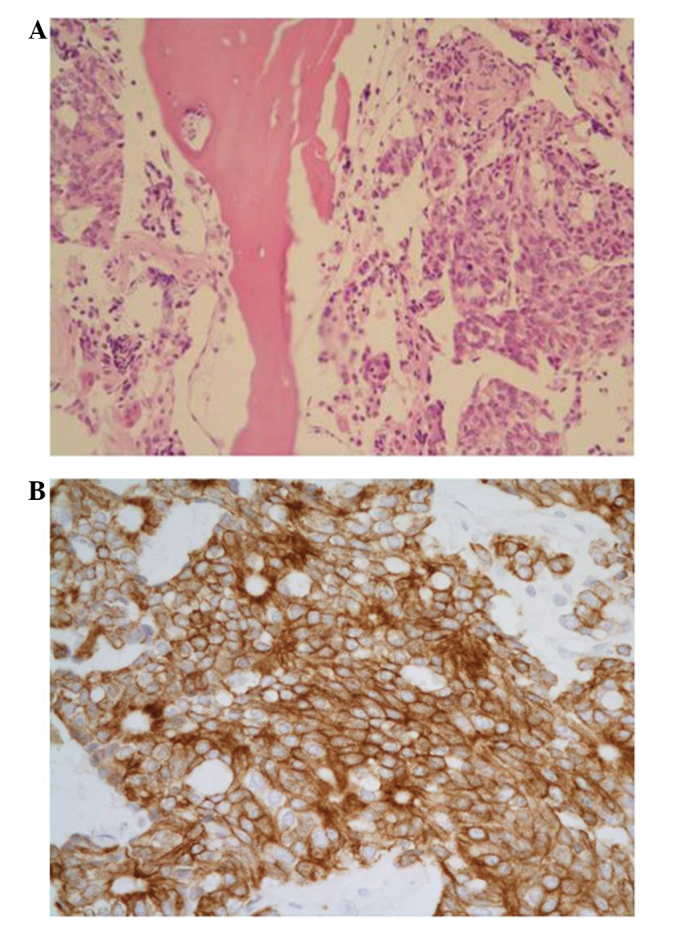
Bone marrow biopsy demonstrating aggregated metastatic cells of colon carcinoma, which were strongly positive for pan-cytokeratin. (A) Hematoxylin and eosin staining; magnification, ×200. (B) Immunohistochemistry for pan-cytokeratin; magnification, ×400.

The final result of the bone marrow biopsy was reported several days later. The overall cellularity was 70–80%, which was hypercellular for the patient’s age, and there was a diffuse infiltration of tumor cells that were negative for leukocyte common antigen and strongly positive for pan-cytokeratin (Fig. 2). The tumor cells infiltrating the bone marrow had the same morphological and immunohistochemical characteristics as the initial colon adenocarcinoma. The patient was finally diagnosed with bone marrow metastasis of colon cancer as an initial site of recurrence.

## Discussion

To the best of our knowledge, the present study is the first to report the bone marrow metastasis of colon cancer as the initial site of recurrence. The clinical course of this case displayed rapid progression with a fatal intracranial hemorrhage associated with severe thrombocytopenia. The patient eventually succumbed despite medical supportive care. The case showed three notable features, namely, the bone marrow metastasis of colon cancer as the first site of recurrence, no other metastatic sites, including skeletal metastasis, and an initial misdiagnosis of drug-induced thrombocytopenia.

Bone marrow metastasis of solid tumors generally develops at the late stage of disease (2) and is accompanied by skeletal metastasis in the majority of cases (1,2). In the mouse model, the route of injection and the number of cancer cells appear to have an effect on the colonization of the bone marrow by the tumor (5). There is evidence suggesting that bone marrow involvement may be a requisite for bone metastasis in this experimental model. Human cases are similar to the animal model, as the solid tumors that frequently involve the bone marrow, for example, lung, breast and prostate cancers, also commonly metastasize to the skeletal system. However, in the present case, the bone marrow was the only site of metastasis and there was no evidence of systemic metastases, including bone metastasis, on abdominopelvic CT scans and simple X-ray. This type of unusual recurrence of the colon cancer was not suspected initially and therefore, this was first cause of delaying the diagnosis of the patient.

Numerous patients visit traditional herbal medicine clinics (Chinese Medicine Clinics) in Korea, particularly those searching for remedies for musculoskeletal problems, as well as general medical conditions. A number of individuals are treated with acupuncture and prescribed herbal medicine with scientifically indefinite ingredients. Drug-induced thrombocytopenia induced by herbal remedies is not an uncommon occurrence in Korea and it represents a significant clinical problem for hematologists. Hematologists should always be aware of whether patients have been taking herbal medicine when they perform the initial assessment of a patient with thrombocytopenia. Drug-induced thrombocytopenia typically induces the sudden onset of thrombocytopenia, which is often severe, and can cause major bleeding and mortality (6,7). Nadir platelet counts are often 20,000/μl. The recovery of thrombocytopenia begins within 1–2 days of the drug being discontinued and recovery is usually complete within a week. In the present study, the patient had a history of taking herbal medicine and exhibited the aforementioned abrupt decrease in platelet count. Therefore, the initial condition was diagnosed as drug-induced thrombocytopenia. This was second cause of the delayed diagnosis of the patient.

There were certain problems with the diagnosis of the present patient, particularly with respect to the bone marrow metastasis of colon cancer, without skeletal metastasis, as the first site of recurrence. Physicians should always suspect the bone marrow involvement of solid tumors in cancer patients with pancytopenia, even when there is no evidence of systemic metastases, including bone metastasis.

In conclusion, for pancytopenia in cancer patients, there should be no delay in performing a bone marrow examination to identify the fatal condition of the present case and manage it properly, although there are other suspected causes.
